# The effectiveness of trendelenburg positioning on the cross-sectional area of the right internal jugular vein in obese patients

**DOI:** 10.12669/pjms.314.7326

**Published:** 2015

**Authors:** Ozkan Onal, Seza Apiliogullari, Alaaddin Nayman, Ali Saltali, Huseyin Yilmaz, Jale Bengi Celik

**Affiliations:** 1 Ozkan Onal, Department of Anesthesia and Intensive Care, Selcuk University Medical Faculty, Konya, Turkey; 2 Seza Apiliogullari, Department of Anesthesia and Intensive Care, Selcuk University Medical Faculty, Konya, Turkey; 3 Alaaddin Nayman, Department of Interventional Radiology, Selcuk University Medical Faculty, Konya, Turkey; 4 Ali Saltali, Department of Anesthesia and Intensive Care, Selcuk University Medical Faculty, Konya, Turkey; 5 Huseyin Yilmaz, Department of General Surgery, Selcuk University Medical Faculty, Konya, Turkey; 6 Jale Bengi Celik, Department of Anesthesia and Intensive Care, Selcuk University Medical Faculty, Konya, Turkey

**Keywords:** Obese patients, Central venous catheterization, Ultrasound, Cross sectional area

## Abstract

**Objective:**

Trendelenburg positioning is a common approach used during internal jugular vein (IJV) cannulation. No evidence indicates that Trendelenburg positioning significantly increases the cross-sectional area (CSA) of the IJV in obese patients. The primary aim of this study was to determine the effectiveness of Trendelenburg positioning on the CSA of the right internal jugular vein assessed with ultrasound measurement in obese patients.

**Methods:**

Forty American Society of Anesthesiologists II patients with body mass index ≥30 kg/m^2^ undergoing various elective surgeries under general endotracheal anesthesia were enrolled. Ultrasound images of the right IJV were obtained in a transverse orientation at the cricoid level. We measured the CSA of the right IJV two different conditions in a sealed envelope were applied in random order: State 0, table flat (no tilt), with the patients in the supine position, and State T, in which the operating table was tilted 20° to the Trendelenburg position.

**Results:**

The change in the CSA of the IJV from the supine to the Trendelenburg position (1.80 cm^2^ vs 2.08cm^2^) was not significantly different. The CSA was paradoxically decreased in 10 of 36 patients when the position changed from State 0 to State T.

**Conclusions:**

Trendelenburg positioning does not significantly increase the mean CSA of the right IJV in obese patients. In fact, in some patients, this position decreases the CSA. The use of the Trendelenburg position for IJV cannulation in obese patients can no longer be supported.

## INTRODUCTION

Venous access is an important issue for surgical patients who are obese.^1^,^2^ A central venous catheter may be necessary if there is difficulty finding viable peripheral venous access in the operative setting.^2^ The right internal jugular vein (IJV) is the vessel often used to place a central venous line. However, IJV catheterization may result in serious complications, including arterial puncture, AV fistula, cardiac tamponade, and even mortality.^3^,^4^ Placement of a central venous catheter may be difficult due to poorly identified neck landmarks in obese patients; in addition, a high body mass index (BMI) has been reported as one of the most important risk factors for complications of IJV cannula placement during the procedure.^2^,^5^-^7^ The optimum condition should be carried out by clinicians before IJV cannulation especially in high-risk obese patients.

The success rate for central venous cannula placement correlates with the cross-sectional area (CSA) of the vein.^5^,^6^,^8^,^9^ A limited number of maneuvers have been attempted to increase the CSA of the IJV in anesthetized patients such as different degrees positive end-expiratory pressure and Trendelenburg positioning.^5^,^6^,^8^,^9^ Most previous studies that evaluated the impact of such maneuvers on the CSA of the IJV were performed in nonobese patients, but the effectiveness and safety of these maneuvers are still being debated.^5^,^6^,^10^,^11^ Our systematic search of the National Library of Medicine (PubMed) did not produce any reports on the effect of the Trendelenburg maneuver in obese patients on the CSA of the IJV.

The aim of this study was to determine the effectiveness of Trendelenburg positioning on the CSA of the right IJV assessed with ultrasound measurement in obese patients who were undergoing various surgeries.

## METHODS

The prospective, controlled study protocol was approved by the Institutional Review Board of the Medical Faculty Hospital, Selcuk University, and written informed consent was obtained from all participants. This study took place in the operating theatre of Selcuk University Hospital from January 2014 to May 2014. We enrolled 40 American Society of Anesthesiologists (ASA) II patients with BMI ≥30 kg/m^2^ who were undergoing various elective surgeries under general endotracheal anesthesia. Patients without previous right IJV cannulation, any previous trauma or surgery involving the neck, or reported limited neck mobility, or pulmonary disease, and patients with coexisting cardiovascular diseases were not included in the study, except for well-controlled hypertension induced by obesity. Patients with stenotic or thrombosed right IJV and hypotension during the ultrasound measurement (systolic blood pressure lower than 90 mmHg) were excluded from the study.

The patients received no premedication. Routine monitoring of blood pressure, electrocardiogram, and pulse oximetry were performed. Induction to anesthesia was performed with fentanyl 100 µg of ideal body weight (IBW), propofol 1.5–2.0 mg/kg of corrected body weight (130% of IBW), and Rocuronium 0.6 mg/kg of IBW.

After endotracheal intubation, anaesthesia was maintained with sevoflurane in oxygen and air (50:50) to keep a target MAC of 0.8 during the image recordings to reduce probable hypotension due to anesthesia without surgical stimulation. Further anesthesia preparation for the surgical procedure was performed after all measurements had been performed. Anaesthesia was maintained with sevoflurane in Vol% depending on age and clinical parameters during the surgery.

The patients were brought to the supine position and the head rotated to 20° to the left side without cervical extension. Ultrasound images of the right IJV were obtained in a transverse orientation at the cricoid level using a LOGIQ Book XP (GE Healthcare, Wauwatosa, WI) with a linear (7.5 MHz) probe. The right IJV was depicted in the middle of the ultrasound image. Probe pressure was kept as low as possible to avoid compression of the IJV.

While the probe was held in the same position, the right IJV was imaged after two different conditions in a sealed envelope were applied in random order: State 0, table flat (no tilt), with the patients in the supine position, and State T, in which the operating table was tilted 20° to the Trendelenburg position. Measurements were made after 2 minutes in each state.

The following measurements were carried out at each position: (1) the CSA of the right IJV, (2) the transverse diameters of the right IJV, and (3) the anteroposterior diameters of the right IJV.

The patients’ demographic data were also collected. We defined a 20% increase in the CSA as clinically significant and patients with an increase of <20% in CSA measurement as “nonresponders”.^9^

**Fig. 1:**
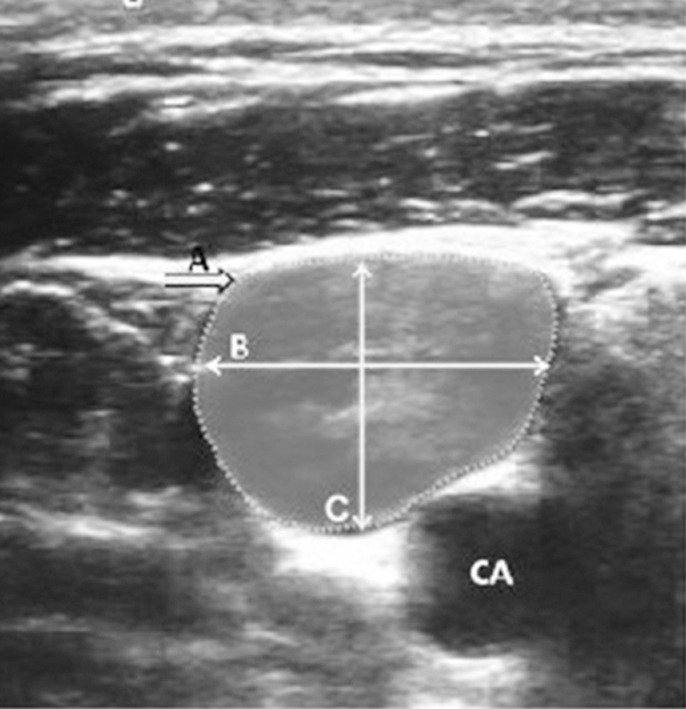
Ultrasound image of the right internal jugular vein and relationship with carotid artery (CA).

Depending on the ventilation, the right IJV CSA varied in size; the image was frozen and stored when the largest area was shown. After the real-time image was frozen, the circumference of the IJV was delineated using an electronic marker, and the CSA of the IJV was calculated using a program preloaded into the ultrasound unit. The transverse and anteroposterior diameters of the right IJV were measured by drawing a line between the furthest two points of the vein wall in the transverse and anteroposterior planes (Fig.1).

Statistical analysis was performed using SPSS version 17.0 (SPSS Inc., Chicago, IL). The data were tested for normality using the Kolmogorov–Smirnov test. The paired sample t-test was used to compare the diameters and CSA changes between State 0 and State T in all patients. We used the Mann–Whitney U test to evaluate the relationships among age, sex, BMI, and CSA regarding changes in CSA. A p-value <0.05 was considered statistically significant.

## RESULTS

Complete data were collected from 36 patients (25 female and 11 male). The patients’ characteristics are shown in Table-I. The examination had to be interrupted due to hypotension in four patients who were excluded from the study. The study measurements are summarized in Fig.2. The CSA, the transverse and anteroposterior diameters of the right IJV between the supine and the Trendelenburg position, were not significantly different. Trendelenburg positioning more than >20% increased (responders) in the CSA of the IJV in only 10 of 36 obese patients. Using the Mann–Whitney U test, we compared the values for the responders (n=10) and the nonresponders (n=26). Twenty-one of 26 female patients were determined as nonresponders (p=0.019, Table-II). The CSA was paradoxically decreased in 10 of 36 patients in Trendelenburg positioning.

**Table-I:** Patients characteristics.

Age (y) mean±SD	43.14±12.37
Sex (n, %) Male/Female	11 (30.6) / 25 (69.4)
BMI (kg/m^2^) mean±SD	45.02±7.31

BMI = Body Mass Index.

**Fig. 2A:**
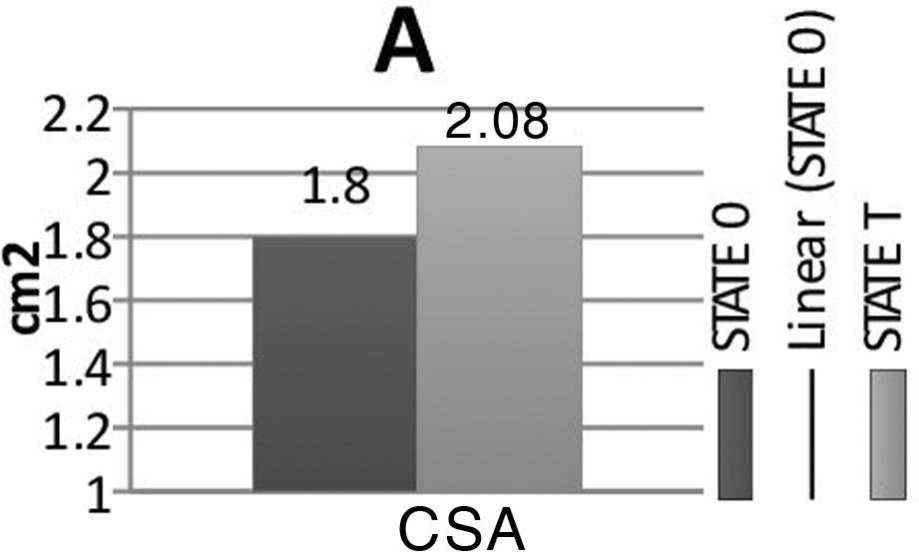
Cross sectional area (CSA) of internal jugular vein in State 0 (Table no tilt) and State T (Table tilted 20° Trendelenburg position).

**Fig. 2B:**
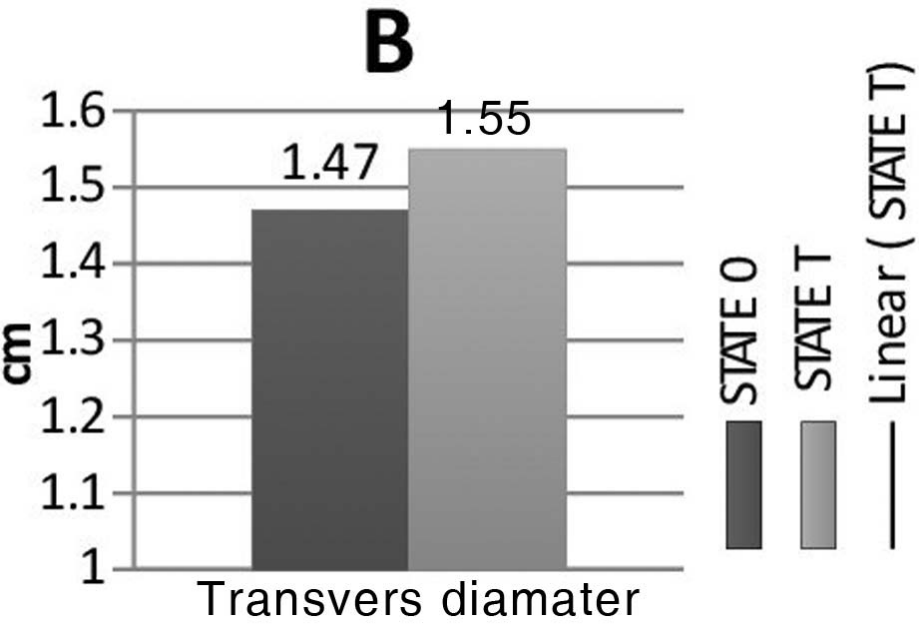
Cross sectional area (CSA) of internal jugular vein in State 0 (Table no tilt) and State T (Table tilted 20° Trendelenburg position).

**Fig. 2C:**
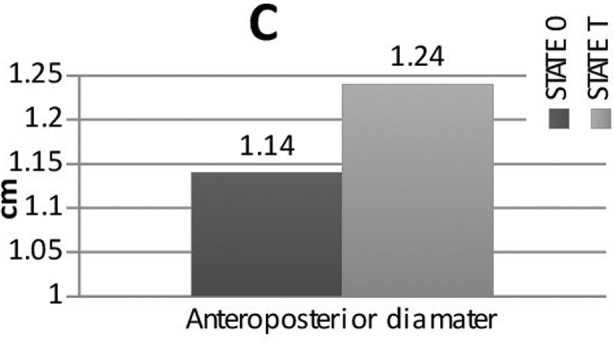
Cross sectional area (CSA) of internal jugular vein in State 0 (Table no tilt) and State T (Table tilted 20° Trendelenburg position).

**Table-II:** Responders and Non-responders

	Responders (n=10)	Non-responders (n=26)
Age (year)	41.80±11.48	43.65±12.87
Male / Female (n, %)	6 (60) / 4 (40)	5 (19.2) / 21 (80.8)*
BMI (kg/m^2^)	45.76±8.00	44.74±7.17
CSA in State 0	1.80±1.32	1.96±1.47

* P= 0.019 when compared with responders.

BMI = Body Mass Index; CSA = Cross Sectional Area

## DISCUSSION

Our work has two major findings: (1) The Trendelenburg position is not effective in increasing the CSA of jugular veins in obese patients, and (2) Trendelenburg positioning actually reduced the CSA in 10 of 36 obese patients.

The Trendelenburg position is the elevation of the pelvis above the horizontal plane in the supine position.^12^ This ritual practice depends on the expectation that increasing the vertical distance between the right atrium and the veins in the neck will raise the pressure in the vein by an amount related to the weight of the blood column.^5^ Factors including cardiac pump function, the jugular venous wall tone, compliance and transmural pressure of IJV, and autonomic tone may affect the CSA of the jugular vein.^5^,^9^ The influence of obesity on cardiovascular systems was well described by Lakhaniand and Fein.^13^ Obesity adversely affects the circulatory system with resultant endothelial dysfunction, which promotes systemic hypertension, coronary artery disease, and vascular calcification.^13^ Morbidly obese patients have 2 to 3 times higher intraabdominal pressure than that of non-obese patients.^14^ To the best of our knowledge, no study has evaluated the effect of Trendelenburg positioning on the CSA of the IJV in obese patients. Moreover, there is no consensus on the effect of the Trendelenburg position regarding increases in the CSA of the IJV in nonobese patients.^5^,^8^,^9^,^11^,^14^-^17^ The expectation of an increase in the CSA under the Trendelenburg position was not observed in the present study. A likely explanation for this is the influence of obesity on the vascular system. Our results are in line with two recently published studies that suggested stopping routine use of Trendelenburg positioning during IJV cannulation in nonobese ICU patients^5^ and dialysis patients.^11^

Wu et al.^11^ conducted an ultrasound study to investigate the efficacy of the use of the Trendelenburg position on the diameters and CSA of the right IJV in patients with chronic kidney disease. Fifty dialysis patients and 40 healthy volunteers were evaluated in that study. The results of the study showed that in contrast to healthy volunteers, there was no enlargement of the right IJV when dialysis patients were in the Trendelenburg position. Nassar et al.^5^ determined the impact of three different positions (15° Trendelenburg, supine, and 15° reverse Trendelenburg) on the CSA of the IJV in critically ill patients. The researchers reported that the CSA was only 17% higher in the Trendelenburg position when compared with the supine position.^5^

The effects of the Trendelenburg position on the diameters of the IJA are largely unknown in obese patients. Woo et al.^18^ reported that the IJV diameter increased by only 10% to 20% when the 10° Trendelenburg tilt was performed in obese Asian patients. In the present study, the Trendelenburg position increased the mean transverse diameter from 1.47 cm to 1.55 cm (5.4% increase) and the anteroposterior diameter from 1.14 cm to 1.24 cm (8.7% increase), but these increases were not significant.

Marcus et al.^9^ evaluated the impact of the Trendelenburg position and positive end-expiratory pressure on the CSA of the IJV. Fifty patients with ASA physical status III scheduled to undergo major cardiothoracic surgery were studied in that study, regardless of BMI. They defined patients with an increase of <20% in the CSA measurement as “nonresponders.” The results showed that all maneuvers increased the CSA of the right IJV compared to the control condition.^9^ The researchers also found a considerable number of “nonresponder” (13 of 50 patients) veins in response to all maneuvers. These nonresponders differ in BMI (27.5 in responders vs 31.8 in nonresponders) but not in age, height, or hemodynamic variables. The authors speculated that higher BMI values are more frequently found in nonresponders because a higher body weight increased compression of the vein. Similar to Marcus et al.^9^, we defined patients with an increase of <20% in CSA measurements as “nonresponders,” and we found that 26 of the 36 obese patients were nonresponders.

Factors predictive of the Trendelenburg position on the CSA of the IJV are largely unknown. Maratea et al.^16^ evaluated the extent of change in the CSA of the right IJV in response to Trendelenburg positioning in 57 anesthetized nonobese patients and its predictive factors. The researchers showed that right IJV dilation in response to the Trendelenburg position was significantly less pronounced, and more variable, in female patients. Most patients (69.4%) in our study were female and had similar CSAs in the supine and Trendelenburg positions. Moreover, most patients defined as nonresponders were also female. We included obese patients regardless of sex differences. Only 11 of 36 patients were male. Further studies that include equal numbers of men and women should be performed to clarify the effect of sex on the CSA of the IJV in obese patients.

Ultrasound guidance has been recommended to increase the success rate of IJV catheterization and decrease the incidence of complications, but it is not always available and complication free.^2^ In the present study, Trendelenburg positioning increased the CSA in 10 of the 36 patients (responders) by >20% but paradoxically decreased the CSA in 10 of the 36 patients. The finding supports that the impact of the Trendelenburg positioning on the CSA is not predictable^5^ and may decrease the CSA and increase complications. Ultrasound guidance is essential to decrease complications in obese patients even when Trendelenburg positioning is used.

There are some limitations in our study. In this study, measurements were obtained in only the 20° Trendelenburg position for State T. Although the angle of the Trendelenburg position has not been shown to affect the CSA of the IJV in nonobese patients, further studies with different angles should be performed in obese patients.^15^ Another limitation is that we obtained images of the IJV after at most two minutes following the change in the patient’s body position from supine to 20° Trendelenburg. Trendelenburg positioning–related hemodynamic effects might change within minutes.^19^ Further studies should be carried out to determine if greater changes occur following a longer period in the Trendelenburg position. Finally, ultrasound measurements can be operator dependent, so we cannot exclude measurement error, and the investigator was not blinded the table positions.

In conclusion, by studying the ultrasound images of the right IJV of obese patients, we determined that the Trendelenburg position does not augment the CSA, on average, compared with the supine position, and may actually decrease the CSA in some patients. Therefore, the Trendelenburg position can no longer be supported for IJV cannulation in obese patients.
